# Increased risk of tuberculosis among foreign-born persons with diabetes in California, 2010–2012

**DOI:** 10.1186/s12889-015-1600-1

**Published:** 2015-03-18

**Authors:** Sarah Ellen Demlow, Peter Oh, Pennan M Barry

**Affiliations:** Tuberculosis Control Branch, Division of Communicable Disease Control, Center for Infectious Diseases, California Department of Public Health, 850 Marina Bay Pkwy, Bldg P/2, Richmond, CA 94804 USA; Division of Infectious Diseases and Vaccinology, School of Public Health, University of California, Berkeley, USA

## Abstract

**Background:**

Diabetes increases the risk of tuberculosis. We sought to identify populations of persons with diabetes in California at further increased risk for tuberculosis to target tuberculosis infection screening and treatment efforts.

**Methods:**

We performed a retrospective population-based analysis of adult (aged ≥18 years) tuberculosis cases reported in California during 2010–2012. Tuberculosis cases with and without diabetes were grouped into regions of birth and stratified by age category. Population estimates were calculated using 2011–2012 California Health Interview Survey data. We calculated tuberculosis disease rate and relative risk of tuberculosis among persons with diabetes stratified by birth location and age group; and the number needed to screen and, if positive, treat for tuberculosis infection to prevent one case of active tuberculosis over 5 years (NNS).

**Results:**

During 2010–2012, among 6,050 adults with active tuberculosis in California, 82% were foreign-born and 24% had diabetes. The overall relative risk for tuberculosis among persons with diabetes was 3.5 (95% confidence interval, 3.3–3.7) with a rate of 21 per 100,000 persons with diabetes. The rate among foreign-born persons with diabetes (141.5/100,000) was almost 12 times greater than among nonforeign-born persons with diabetes (12.0/100,000). The NNS was 7,930 among all adults, 2,740 among adults with diabetes, 1,526 among all foreign-born adults, and 596 among foreign-born adults with diabetes.

**Conclusions:**

In California, foreign-born persons with diabetes had significantly elevated rates of active tuberculosis. Focusing tuberculosis infection screening and treatment efforts on foreign-born persons with diabetes may be a feasible and efficient way to make progress toward tuberculosis elimination in California.

**Electronic supplementary material:**

The online version of this article (doi:10.1186/s12889-015-1600-1) contains supplementary material, which is available to authorized users.

## Background

Tuberculosis (TB) remains a significant public health threat in the United States and California with more than 2,000 reported cases in California annually. Applying national estimates of the prevalence of TB infection to the California population, approximately 2.5 million Californians are infected with *Mycobacterium tuberculosis* and thus are at risk for progression to active disease in the future [1,2]. Annual diabetes prevalence in California has risen from 4.7% of the population in 1994 to almost 9% in 2010 [3]. Because diabetes has been shown to triple a person’s risk of active TB disease and may confer poorer TB treatment outcomes, the global convergence of the TB and diabetes epidemics has the potential to create significant challenges for TB control [4-15]. California may be particularly subject to these changing trends in diabetes and TB epidemiology in part because it is a frequent destination for immigrants to the United States from regions of the world with high TB incidence. In 2012, the proportion of the general California population that was born outside the United States was more than twice the national average [16]. Despite declines, the 2012 TB case rate in California remained more than 1.5 times higher than the national average of 3.2 cases per 100,000 [17,18]. TB cases that are the result of reactivation of latent TB infection (LTBI) (much of which was acquired abroad) make up approximately 75–80% of TB cases in the United States [19,20]. These cases are likely to be preventable by screening for and treating LTBI, but the combination of a difficult-to-complete regimen and imperfect tests for LTBI has limited the feasibility of finding and treating more than a small fraction of persons with LTBI [21]. However, mathematical modeling indicates that increased screening and treatment for LTBI would have the largest impact on lowering case rates in the United States [22]. In order to make progress on reducing TB, more efficient ways to identify and treat patients at risk for progression to active TB are needed. An important TB control strategy might be to prevent TB cases among persons with higher risk, such as those with diabetes. However, because TB rates vary substantially among subpopulations in California, prevention efforts among all persons with diabetes is not likely to be feasible or cost effective.

We aimed to identify subpopulations in California where the convergence of TB and diabetes warrants increased screening and treatment.

## Methods

To investigate the risk of TB among persons with diabetes, we conducted a retrospective population-based study of adult (aged ≥18 years) noninstitutionalized TB cases reported to the California Department of Public Health (CDPH) TB Registry during the three year period 2010–2012. Since 2010, the California TB Registry has included information on diabetes mellitus history – either type I or type II, but not borderline, pre-diabetes, or gestational diabetes. This information is gathered by local TB control programs from medical records or a health care provider. Self-report of diabetes is not considered an acceptable source of diabetes status [23]. We also queried the TB Registry for patient age, gender, country of birth and primary reason for the medical evaluation leading to TB diagnosis. Persons diagnosed in a long term care facility or a correctional facility were excluded from analysis. The reason for evaluation was one of the following: TB symptoms, abnormal chest radiograph consistent with TB, contact investigation, targeted testing, occupational health care worker screening, other employment or administrative screening, immigration medical exam, or incidental laboratory result [23]. Race/ethnicity was reported by local TB control programs based on patient self-report according to United States government standards [24].

Because race/ethnicity correlates closely with location of birth and examining only race/ethnicity could mask important differences among persons of the same race/ethnicity born in different locations, we chose to analyze data according to location of birth, rather than race/ethnicity.

Using data on country of birth, we grouped TB cases with and without diabetes into the following seven locations of birth: United States and Canada, Latin America (including Mexico), Southeast Asia/Pacific Islands (SEA/PI; e.g., Philippines, Vietnam), East Asia (e.g., China, Korea), Europe, South Asia (e.g., India), and Africa/Middle East. Persons born outside of the United States or Canada were considered foreign-born.

There is no statewide diabetes surveillance registry in California. The population with diabetes was estimated using data from the 2011–2012 California Health Interview Survey (CHIS) using the variable “ever diagnosed with diabetes [25]”. CHIS is a biannual population-based telephone survey (reaching both landline and cellular phones), whose sample is representative of California’s noninstitutionalized population living in households. Diabetes estimates were for adults aged ≥18 years, and included all forms of diabetes except gestational diabetes, pre-diabetes and borderline diabetes. The demographic characteristics from CHIS included in our analysis were age group and location of birth.

Because individual level data on age and location of birth for persons without TB were not available, we were unable to perform a multivariable analysis. Instead, we took a stratified analysis approach to control for these variables simultaneously. We stratified data by age group (18–44, 45–64, 65–74 and 75 years and over). For each age group/location of birth combination, we calculated the average annual TB incidence (TB rate, expressed as cases per 100,000 population) for the population without diabetes and for the population with diabetes, and the relative risk of TB among persons with diabetes compared with those without diabetes using TB registry data for the numerator and CHIS data for the denominator. Ninety-five percent confidence intervals (95% CI) were calculated using STATA 11.0 (StataCorp, College Station, Texas). We considered *P* values <0.05 as significant using 2-sided testing. We applied the *χ*^2^ test for comparisons between the group of TB cases with diabetes and the group of TB cases without diabetes.

The number of persons needed to screen and, if positive, treat for TB infection to prevent one case of active TB (NNS) over a five year period was calculated as being equal to the inverse of the absolute risk reduction among those screened compared with those not screened [26]. NNS was calculated using TreeAge Pro 2013 (Williamstown, MA) with inputs drawn from published literature [1,2,27-29] and from the estimated relative risk of TB conferred by diabetes calculated in the current analysis. Parameters included in the NNS calculation included prevalence of LTBI, risk of progression to active TB over five years, LTBI screening test sensitivity, efficacy of LTBI treatment, and proportion of persons with LTBI who start and complete treatment (see Additional file 1: Table S1 for inputs and Figure S1 for decision tree). Sensitivity analyses were performed across a range of estimates for prevalence of LTBI and rate of progression to active TB over five years.

California state law requires TB cases to be reported to local public health departments and to CDPH (California Code of Regulations Title 17 §2500, §2502, and §2505). This analysis was conducted using case report data and is part of CDPH’s mandate to routinely collect and analyze surveillance data for public health purposes. Therefore, consent was not obtained and this analysis did not require human subjects review, according to CDPH policy.

Additional aggregate supporting data is available from the authors upon request.

## Results

During 2011–2012 there were an estimated 27,797,000 noninstitutionalized adults aged ≥18 years in California, of whom approximately 2,322,000 (8.4%) had diabetes. During 2010–2012, 1,463 (24%) of the 6,050 active TB cases in this population had diabetes. Among both TB cases and the general adult population in California, persons with diabetes were more likely to be male, older than 44 years, and foreign-born (Table 1).

**Table 1:** **Demographic characteristics of adult populations with TB and diabetes ― California 2010–2012**

**Characteristic**	**CA adult population with diabetes** ^**a**^		**CA adult population without diabetes** ^**a**^		**TB cases with diabetes** ^**b**^		**TB cases without diabetes** ^**b**^	
	**n**	**(%)**	**n**	**(%)**	**n**	**(%)**	**n**	**(%)**
All	2,322,000	(100)	25,475,000	(100)	1,463	(100)	4,587	(100)
Gender^c^								
Male	1,174,000	(50.6)	12,368,000	(48.6)	939	(64.2)	2,592	(56.5)
Age group^c^								
18–44	349,000	(15.0)	13,715,000	(53.8)	200	(13.7)	2,061	(44.9)
45–64	1,129,000	(48.6)	8,200,000	(32.2)	693	(47.4)	1,414	(30.8)
65–74	498,000	(21.4)	1,910,000	(7.5)	275	(18.8)	509	(11.1)
≥75	346,000	(14.9)	1,649,000	(6.5)	295	(20.2)	603	(13.1)
Race and/or ethnicity^c^								
White, Non-Hispanic/Latino	866,000	(37.3)	11,228,000	(44.1)	67	(4.6)	423	(9.2)
Hispanic/Latino	945,000	(40.7)	8,571,000	(33.6)	573	(39.2)	1,526	(33.3)
Asian/Pacific Islander	283,000	(12.2)	3,672,000	(14.4)	774	(52.9)	2,318	(50.5)
Black, Non-Hispanic/Latino	178,000	(7.7)	1,388,000	(5.4)	46	(3.1)	311	(6.8)
American Indian	17,000	(0.7)	105,000	(0.4)	3	(0.2)	9	(0.2)
Other	33,000	(1.4)	511,000	(2.0)	---	---	---	---
Location of Birth^c^								
United States or Canada	1,405,000	(60.6)	17,127,000	(67.5)	168	(11.5)	844	(18.4)
Latin America^d^	615,000	(26.5)	4,738,000	(18.7)	492	(33.7)	1,246	(27.2)
Southeast Asia/Pacific Islands^e^	131,000	(5.7)	1,073,000	(4.2)	593	(40.6)	1,423	(31.1)
East Asia^f^	86,000	(3.7)	1,045,000	(4.1)	114	(7.8)	517	(11.3)
Europe^g^	36,000	(1.6)	666,000	(2.6)	14	(1.0)	70	(1.5)
South Asia^h^	24,000	(1.0)	387,000	(1.5)	55	(3.8)	302	(6.6)
Africa/Middle East^i^	21,000	(0.9)	343,000	(1.4)	24	(1.6)	174	(3.8)

^a^Source: California Health Interview Survey, 2011–2012.

^b^Source: California TB case registry, 2010–2012. Excludes patients who were institutionalized (i.e., incarcerated, resident of a long-term care facility) at the time of TB diagnosis.

^c^Chi- square p-value comparing the “TB cases with diabetes” group to “TB cases without diabetes” < .001.

^d^Mexico, Central America, South America and the Caribbean.

^e^Philippines, Vietnam, Cambodia, Laos, Myanmar, Indonesia, Pacific Islands, Thailand, Malaysia, Australia, Singapore.

^f^China, Korea, Taiwan, Hong Kong, Japan, Mongolia.

^g^Including Russia, Armenia, Azerbaijan, Turkey.

^h^India, Pakistan, Nepal, Bangladesh, Bhutan, Sri Lanka.

^i^All African countries, and Iran, Afghanistan, Iraq, Lebanon, Saudi Arabia, Syria, Yemen, Jordan.

Region of origin numbers may not sum to population totals because of limitation of the available CHIS data.

The overall TB rate among persons with diabetes in California was 21 cases per 100,000 yielding a relative risk of 3.5 (95% CI, 3.3–3.7; Table 2). The rate was higher among persons with diabetes compared with persons without diabetes across all strata of age and location of birth. Foreign birth and diabetes had a multiplicative effect on TB rate with diabetic foreign-born persons having an overall TB rate of 142/100,000, almost 12 times greater than among United States or Canadian-born persons with diabetes (12/100,000). This relationship held after stratifying by age with rates of TB 22–40 times higher among foreign-born persons with diabetes than persons born in the United States or Canada without diabetes. Among persons with diabetes, the highest reliable TB rates occurred among persons born in Southeast Asia and the Pacific Islands (SEA/PI) with age-specific TB rates of 117–239/100,000. Persons with diabetes born in Latin America also had elevated TB rates with a range of 23–68/100,000. Persons born in the United States or Canada had lower rates of TB among both (range: 1.8–8.2) persons with and without diabetes (range: 1.5–2.7).

**Table 2:** **Average annual rate and relative risk of tuberculosis by diabetes status, stratified by age group and location of birth—California, 2010–2012**

**Location of birth**	**Age group**	**TB case rate per 100,000 adults with diabetes**	**TB case rate per 100,000 adults without diabetes**	**Relative risk (95% confidence interval)**
All	All	21.0	6.0	3.5 (3.3–3.7)
United States or Canada	18-44	8.2	1.5	5.5 (4.1–7.5)
	45-64	4.0	1.7	2.4 (1.8–3.1)
	65-74	*1.8*	1.6	1.2 (0.7–2.0)
	75+	3.4	2.7	1.3 (0.8–1.9)
Foreign-born	18-44	33.0	11.9	2.8 (2.4–3.3)
	45-64	41.9	13.4	3.1 (2.8–3.5)
	65-74	45.2	32.9	1.4 (1.2–1.6)
	75+	110.5	51.6	2.1 (1.8–2.5)
Latin America	18-44	23.0	7.4	3.1 (2.5–3.9)
	45-64	24.1	7.5	3.2 (2.7–3.8)
	65-74	25.4	24.3	1.0 (0.8–1.4)
	75+	68.4	34.6	2.0 (1.5–2.6)
Southeast Asia/Pacific Islands	18-44	117.0	34.1	3.4 (2.6–4.6)
	45-64	144.7	42.9	3.4 (2.9–3.9)
	65-74	134.6	68.0	2.0 (1.6–2.5)
	75+	239.1	130.5	1.8 (1.4–2.3)
East Asia	18-44	*18.9*	11.0	1.7 (0.6–4.6)
	45-64	34.8	12.1	2.9 (2.0–4.1)
	*65-74*	*24.7*	30.4	0.8 (0.5–1.4)
	75+	106.1	63.5	1.7 (1.2–2.3)
South Asia	18-44	*48.8*	24.5	2.0 (0.9–4.5)
	45-64	50.7	15.2	3.3 (2.0–5.5)
	65-74	*87.4*	90.6	1.0 (0.4–2.2)
	75+	*582.0*	553.7	1.1 (0.6–1.9)
Africa/Middle East	18-44	*239.5*	15.0	16.0 (7.0–36.5)
	45-64	*17.7*	12.3	1.4 (0.7–3.1)
	65-74	*37.2*	*33.4*	1.1 (0.4–3.4)
	75+	*109.8*	72.5	1.5 (0.6–3.7)
Europe	18-44	*14.1*	2.6	5.5 (1.3–23.1)
	45-64	*18.3*	3.2	5.8 (2.3–14.4)
	65-74	*12.0*	*5.0*	2.4 (0.8–7.7)
	75+	*6.9*	*6.8*	1.0 (0.2–4.4)

Italicized numbers contain estimates based on a numerator of <20 and may be unstable.

Despite large differences in TB rates by location of birth, the relative risk of TB conferred by diabetes was relatively consistent across locations of birth but with higher relative risks observed among the two youngest age categories: range of 2.4–5.5 compared with 1.2–2.1 among the oldest two age categories.

The number of persons needed to be screened and treated for TB infection to prevent one case of active TB varied by population (Table 3). In the overall California population of adults, 7,930 persons would need to be screened and treated. Among all persons with diabetes, the number drops to 2,740 with further decreases to 1,526 among foreign-born persons, and to 596 among foreign-born persons with diabetes. Among subgroups of persons with diabetes by region of birth, NNS was lowest among persons born in regions of Asia and in Latin America.

**Table 3:** **Estimates of the number needed to screen and treat (NNS) for TB infection to prevent one case of active TB in the subsequent 5 years among groups by demographic and diabetes status in California**

**Target population of TB screening and treatment**	**NNS base case**	**TB Infection prevalence** ^**a**^		**Rate of progression to active TB** ^**a**^	
		**High**	**Low**	**High**	**Low**
All adults	7,930	6,667	10,000	3,846	14,286
All foreign-born adults	1,526	1,136	2,128	763	2,941
All adults with diabetes	2,740	2,222	3,448	1,370	5,556
U.S.-born adults with diabetes	9,551	7,143	12,500	4,762	20,000
Foreign-born adults with diabetes	596	442	826	298	1,163
Foreign-born adults with diabetes by location of birth					
Latin America	741	526	877	370	1,493
Southeast Asia/Pacific Islands	296	206	361	148	585
East Asia	372	260	452	186	730
South Asia	335	234	408	168	662
Europe	2,221	1,493	3,448	1,111	4,545
Africa/Middle East	384	270	562	192	769

^a^High and low indicate the range of NNS using a range of TB infection prevalence and Rate of progression to active disease. See Additional file 1: Table S1 for inputs and ranges used.

The primary reason TB patients presented for TB evaluation varied by diabetes status. Among patients with TB and diabetes, 70.6% were evaluated because of symptoms consistent with TB compared with 64.6% of cases without diabetes (p < 0.001). When persons evaluated for TB because of an abnormal chest radiograph (done for reasons other than suspicion for TB) and incidental laboratory result are considered, a total of 95.8% of TB cases with diabetes compared with 91.0% of cases without diabetes were detected outside of a program designed to identify TB disease or infection (p < 0.001). In contrast, there was no difference in the proportion of cases identified in targeted testing programs by diabetes status, though these numbers were small (0.8% vs. 0.9%, p = 0.74).

## Discussion

In this population-based study of the risk of TB among persons with diabetes in California during 2010–2012, we found that both the increased TB rate and the TB risk among persons with diabetes varied by birth location and age. Foreign-born persons with diabetes had elevated rates of TB compared to both foreign-born persons without diabetes and persons with diabetes born in the United States or Canada. Foreign birth and diabetes had a multiplicative effect on TB case rate. Similar to other studies, we also found that diabetes was common among persons with TB and that diabetes increases the risk of TB disease 3.5 times compared to those without diabetes [4-15]. Persons with diabetes who were born in SEA/PI had particularly high rates of TB. Our NNS analysis showed that fewer foreign-born persons with diabetes would need to be screened and treated to prevent one case of active TB than other populations, suggesting that this group may be an appropriate population on which to focus TB screening efforts. Furthermore, we found that a high proportion of TB patients including those with diabetes came to be diagnosed with TB only after seeking medical attention for TB-related symptoms, or after having unexpected laboratory or radiographic findings. Few TB cases among persons with diabetes were identified by targeted screening programs for persons with increased risk for TB, suggesting that persons with diabetes are not currently being effectively screened for TB infection. These findings could form the basis for focusing increased efforts on screening for and treating TB infection among foreign-born patients with diabetes, particularly those born in Southeast Asia or the Pacific Islands.

Our analysis shows that the number of persons needed to be screened and, if positive, treated to prevent one case of TB over a five year period is smallest among foreign-born persons with diabetes. In this group, the NNS is within range of other routinely practiced screening activities to prevent death such as blood pressure monitoring, hemoccult testing, mammograms, and others [26,30,31]. Implementing screening and treatment among foreign-born persons with diabetes might be a way to increase the efficiency of screening and treatment programs. Because many patients with diabetes are seen by healthcare providers regularly, working with programs and clinics that provide care to these patients may be feasible but may require development of additional protocols and procedures. Additionally, implementation of the 12 once-weekly doses of isoniazid and rifapentine regimen for TB infection, [32,33] may provide new opportunities for treatment in this group.

A 2011 study that modeled the cost effectiveness of screening for TB infection among various at-risk populations in the United States found that screening solely based on diabetes status was not considered cost effective. However, foreign-born persons aged 25–44 years living in the United States for greater than 5 years independent of diabetes status was one of the most cost-effective groups to screen [28]. Therefore, focusing on patients with diabetes who are foreign-born may further increase cost-effectiveness of TB screening.

Other significant findings from our analysis include the observation that the increased risk of TB conferred by diabetes varied by age group and that in general the lowest increase in risk was found in the oldest age group. Conversely, persons aged 18–44 years in many birth regions had some of the highest relative risks for TB among persons with diabetes. This phenomenon has been observed in a similar analysis of TB and diabetes risk in Mexico [34]. A possible explanation for this phenomenon is that the age-related increased risk of TB among older persons without diabetes is greater than among persons with diabetes. Despite the increased relative risk among younger persons with diabetes, the oldest persons with diabetes had higher rates of TB than the youngest, and so older patients with diabetes should be included in TB screening efforts.

The only previous study to assess TB-diabetes comorbidity in California was published in 1997 and analyzed discharge data from civilian hospitals in 1991 [5]. Since then, California demographics and TB case reporting have changed significantly enabling more accurate TB risk analysis using statewide surveillance data. In a more recent nationwide Australian cohort study from 2012, researchers examined the risk of TB disease by diabetes status and found significantly elevated risk even after adjusting for incidence of TB in a person’s birth country (adjusted RR =1.48). The difference in magnitude between that study and our study may be related to higher prevalence of diabetes in key populations as well as possibly differences in health system structure and prevention to adequately address diabetes in the community [35].

The particularly high rate of TB among persons with diabetes born in SEA/PI was striking. The majority of cases in California in the SEA/PI-born subgroup were born in the Philippines (1106/2065; 54%), where the estimated TB incidence is high compared to other countries in the region (270 cases per 100,000) [36]. Additionally, the prevalence of type 2 diabetes among Filipino immigrants to the United States is high, despite the absence of high rates of obesity [37-40]. As high body mass index has been shown to be protective in many studies against some communicable diseases including TB [41] we may be observing higher relative risks of TB among Filipino-born persons with diabetes because the protective effect of high body mass index observed among persons with diabetes born in other regions is less apparent in the Filipino-born population.

There are several potential limitations to this study. The data in this study are cross-sectional and thus cannot assess temporal order concerning the onset of diabetes or TB disease. However, studies that have had the capacity to assess the relative timing of TB and diabetes diagnoses have also identified increased risk of TB among patients with diabetes, and a meta-analysis found that these studies reported higher relative risks than studies that were not able to establish the order of diagnosis [12,13]. We did not have individual-level data for persons who did not have TB and thus could not construct a multivariable model to generate an adjusted relative risk for TB among persons with diabetes. Nevertheless, rates among subgroups are more useful for focusing screening programs and so the stratified analysis presented should be helpful to clinicians and policy-makers. Diabetes status among those without TB is self-reported and may be dependent on one’s ability to access healthcare to receive a diabetes diagnosis. This may have caused us to overestimate the rate of TB among persons with diabetes, particularly among immigrant communities with potentially less access to care. A recent study of diabetes prevalence among Kaiser Permanente Northern California patients found an overall diabetes prevalence (8.9%) similar to that observed in our study and elevated diabetes rates among racial/ethnic subgroups [40]. These findings among persons with access to care may support our estimates, though that study did not assess prevalence by place of birth. Our calculation of NNS is limited by lack of specific information regarding variations in TB infection prevalence and rate of progression among the subgroups in our analysis. This necessitated using published estimates from national studies and applying estimates of TB infection prevalence and rate of progression for racial/ethnic groups to groups by region of birth (e.g., estimates for foreign-born Asian persons were applied to persons born in SEA/PI, East Asia, and South Asia). This likely masked some of the differences observed in the TB rate between subgroups in our analysis. Last, we did not have data on other factors that could affect the TB-diabetes relationship such as body mass index, duration, severity, or treatment of diabetes, so we were not able to calculate TB rates or relative risks for TB by these factors.

## Conclusion

Our analysis identifies the significant contribution that diabetes is likely playing in fueling current and future TB cases in California. However, because the TB rate is concentrated among foreign-born persons with diabetes, our results point to an opportunity to focus TB prevention resources where they might have the most impact. Implementing these results may require changes in TB infection screening guidelines and performance measures at both the state and national level and among both the TB and diabetes communities. In particular, clear recommendations to screen and treat foreign-born persons with diabetes for TB infection are needed.

## Additional file

Additional file 1: Table S1. Parameters and inputs of the effectiveness (number needed to screen and, if positive, treat for TB infection) analysis. **Figure S1.** TB screening decision tree used to calculate number needed to screen and, if positive, treat for TB infection.

## Abbreviations

CDPH: California Department of Public Health
CHIS: California Health Interview Survey
NNS: Number Needed to Screen
RR: Relative Risk
TB: Tuberculosis
