# Supplementary material for: Increased risk of tuberculosis among foreign-born persons with diabetes in California, 2010–2012
Source: BMC Public Health. 2015 Mar 18;15:263. doi: 10.1186/s12889-015-1600-1 (PMC4381455; doi:10.1186/s12889-015-1600-1)
Supplement: Additional file 1: Table S1. — Parameters and inputs of the effectiveness (number needed to screen and, if positive, treat for TB infection) analysis. Figure S1. TB screening decision tree used to calculate number needed to screen and, if positive, treat for TB infection. [file 12889_2015_1600_MOESM1_ESM.docx]

Additional file 1

Table S1: Parameters and inputs of the effectiveness (number needed to screen and, if positive, treat for TB infection) analysis.

|  |  | Range |  |  |
| --- | --- | --- | --- | --- |
| Parameter | Base | Low | High | Reference(s) |
| Prevalence of TB infection |  |  |  |  |
| All | 4.2% | 3.3% | 5.2% | Bennett et al., 2008 |
| by race / ethnicity only (disregarding nativity) |  |  |  |  |
| White (non-Hispanic/Latino) | 1.9% | 1.3% | 2.9% | Bennett et al., 2008 |
| Black (non-Hispanic/Latino) | 7.0% | 5.3% | 9.1% | " |
| Hispanic/Latino | 9.9% | 7.6% | 11.5% | Shea et al., 2014; Bennett et al., 2008 |
| Asian | 11.4% | 8.0% | 15.0% | Shea et al., 2014; range: assumption |
| by nativity, and race/ethnicity |  |  |  |  |
| U.S. born | 1.8% | 1.3% | 2.4% | Bennett et al., 2008; Shea et al., 2014 |
| Foreign-born | 18.7% | 13.5% | 25.2% | " |
| White (non-Hispanic/Latino) | 17.9% | 11.4% | 26.8% | " |
| Black (non-Hispanic/Latino) | 20.0% | 13.7% | 28.4% | " |
| Hispanic/Latino | 17.7% | 15.0% | 25.0% | Shea et al., 2014; range: assumption |
| Asian | 24.4% | 20.0% | 35.0% | " |
| Sensitivity of TB screening test | 89.0% |  |  | Linas et al., 2011 |
| Proportion of screened persons with TB infection who start treatment | 90.0% |  |  | " |
| Proportion of persons starting treatment who complete | 82.0% |  |  | Sterling et al., 2011 |
| Risk of progression from infection to disease in the absence of treatment (5 years) |  |  |  |  |
| All | 0.42% | 0.21% | 0.84% | Shea et al., 2014; range: assumption |
| by race/ethnicity only (disregarding nativity) |  |  |  |  |
| White (non-Hispanic/Latino) | 0.23% | 0.11% | 0.45% | " |
| Black (non-Hispanic/Latino) | 0.45% | 0.23% | 0.90% | " |
| Hispanic/Latino | 0.45% | 0.22% | 0.89% | " |
| Asian | 0.95% | 0.48% | 1.90% | " |
| by nativity, and race/ethnicity |  |  |  |  |
| U.S.-born | 0.41% | 0.21% | 0.82% | " |
| Foreign-born | 0.49% | 0.25% | 0.98% | " |
| White (non-Hispanic/Latino) | 0.12% | 0.06% | 0.23% | " |
| Black (non-Hispanic/Latino) | 1.00% | 0.50% | 2.00% | " |
| Hispanic/Latino | 0.43% | 0.22% | 0.86% | " |
| Asian | 0.69% | 0.35% | 1.38% | " |
| Risk reduction of treatment for latent TB infection | 90% |  |  | Linas et al., 2011 |
| Relative risk of active TB for persons with diabetes |  |  |  |  |
| All | 3.5 |  |  | Derived from Table 1 |
| by race/ethnicity only (disregarding nativity) |  |  |  |  |
| White (non-Hispanic/Latino) | 2.1 |  |  | " |
| Black (non-Hispanic/Latino) | 1.2 |  |  | " |
| Hispanic/Latino | 3.4 |  |  | " |
| Asian | 4.3 |  |  | " |
| by nativity and region of origin |  |  |  | " |
| U.S.-born | 2.4 |  |  | " |
| Foreign-born | 3.1 |  |  | " |
| Latin American | 3.0 |  |  | " |
| Southeast Asian/Pacific Islander | 3.4 |  |  | " |
| East Asian | 2.7 |  |  | " |
| South Asian | 3.0 |  |  | " |
| European | 3.7 |  |  | " |
| African/Middle Eastern | 2.2 |  |  | " |

Figure S1: TB screening decision tree used to calculate number needed to screen and, if positive, treat for TB infection.


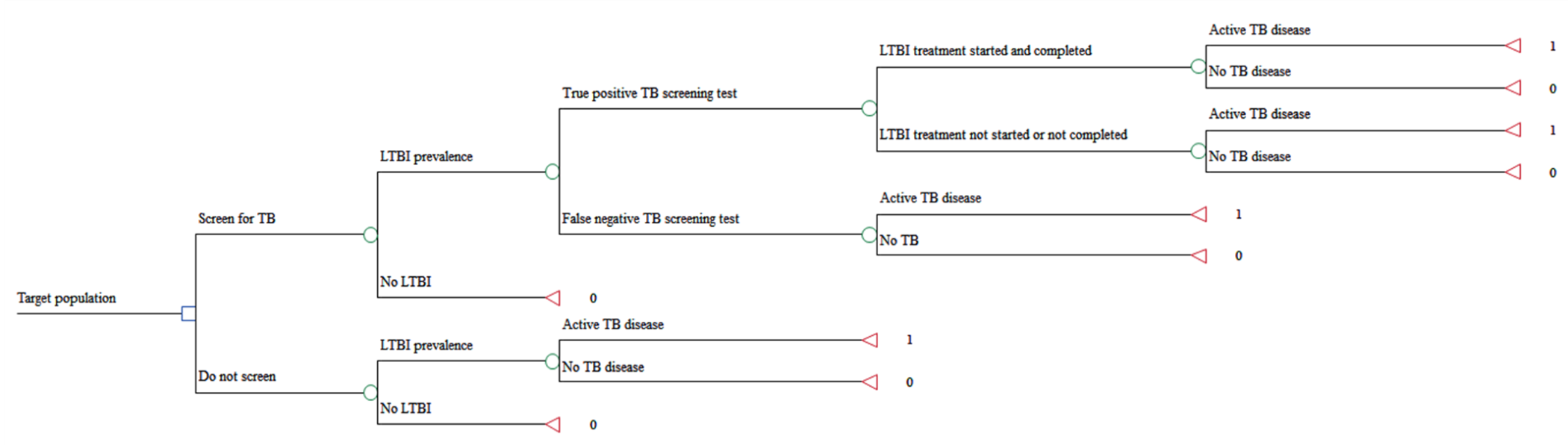


Binary outcome 1=Active TB disease, 0=No active TB disease. The square denotes a decision node. Circles denote chance nodes.

Triangles depict terminal nodes. Descriptions are above the branch lines. Probabilities to the right of each chance node sum to 1, therefore # is 1 minus the probability at that node.
